# ALS-linked TDP-43 mutations interfere with the recruitment of RNA recognition motifs to G-quadruplex RNA

**DOI:** 10.1038/s41598-023-33172-5

**Published:** 2023-04-12

**Authors:** Akira Ishiguro, Akira Ishihama

**Affiliations:** Research Center for Micro-Nano Technology, Hosei University, Midori-cho 3-11-15, Koganei, Tokyo 184-0003 Japan

**Keywords:** RNA metabolism, Molecular biology, Neuroscience, Diseases of the nervous system

## Abstract

TDP-43 is a major pathological protein in sporadic and familial amyotrophic lateral sclerosis (ALS) and mediates mRNA fate. TDP-43 dysfunction leads to causes progressive degeneration of motor neurons, the details of which remain elusive. Elucidation of the molecular mechanisms of RNA binding could enhance our understanding of this devastating disease. We observed the involvement of the glycine-rich (GR) region of TDP-43 in the initial recognition and binding of G-quadruplex (G4)-RNA in conjunction with its RNA recognition motifs (RRM). We performed a molecular dissection of these intramolecular RNA-binding modules in this study. We confirmed that the ALS-linked mutations in the GR region lead to alteration in the G4 structure. In contrast, amino acid substitutions in the GR region alter the protein structure but do not void the interaction with G4-RNA. Based on these observations, we concluded that the structural distortion of G4 caused by these mutations interferes with RRM recruitment and leads to TDP-43 dysfunction. This intramolecular organization between RRM and GR regions modulates the overall G4-binding properties.

Amyotrophic lateral sclerosis (ALS) is a progressive neurodegenerative disorder characterized by the selective death of motor neurons in the motor cortex, brainstem, and spinal cord^1,2^. Approximately 10% of the cases involve genetic mutations, whereas the remaining cases are considered sporadic due to unknown causes^3^. Currently, more than 50 causative or potentially related genes have been identified, of which the majority encode RNAs and RNA-binding proteins (RBPs)^4,5^. Therefore, identifying and analyzing the state of RNA molecules common to all is essential. Indeed, without exception, all these RNAs and RBPs are associated with G-quadruplexes (G4) and are components of ribonucleoprotein (RNP) granules assembled through liquid–liquid phase separation (LLPS)^6^ (Table S1). This is surprising because there are not many RBPs that specifically recognize G4^7,8^. Since these gene mutations are thought to be involved in the onset of ALS prominent, the research on the structure and function of these RNAs and proteins is essential for understanding ALS pathogenesis^9^.

TDP-43 (43 kDa TAR DNA-binding protein, encoded by *TARDBP*) is an ALS-linked RBP^4^. The well-studied TDP-43 is a major disease-related protein involved in the pathogenesis of ALS, leading to form abnormal intraneuronal inclusions detected in more than 90% of patients^10^. Mutations in the TDP-43 gene have also been identified in familial and sporadic ALS patients^3,11^. Previously, we identified that TDP-43 binds to G4-containing mRNAs and transports them to distal neurites for local translation^12^. G4 is a higher-order DNA/RNA structure consisting of two or more guanine tetrads originating from four guanine bases assembled in a square planar arrangement via a Hoogsteen hydrogen bond scheme^13^. G4-binding RBPs may play essential roles in the regulation of mRNA transport and function, and the dysregulation of RBPs is feared to cause neurological disorders^14,15^. However, no detectable destabilization was observed for mutant TDP-43 proteins^16–18^, thus dysfunction of TDP-43 might be the cause of the disease onset.

Herein, we performed the molecular dissection of TDP-43. It contains two RNA recognition motifs (RRM) and a C-terminal glycine-rich (GR) region and forms a homodimer under physiological cellular conditions^19^. Most of the disease-associated amino acid substitutions are located within the uncharacterized GR region^3^. With the use of TDP-43 with ALS-linked mutations in RRM or GR region, we found a reduction of G4-mRNA transport into distal neurites for local translation, suggesting the participation of both regions in binding and function^12,20^. We then speculated the involvement of multiple modules in the recognition of G4-RNA. We confirmed that the RRM and GR regions bind in concert to parallel-stranded G4 conformations^21^.

To clarify the roles of each RRM and GR region in the recognition of G4-RNA, we investigated the influence of ALS-linked TDP-43 mutations on the G4-RNA binding activity using these RNA-binding module segments. The mutations in GR segment alone induced the conformation alteration of G4-RNA while it showed the effects on G4-RNA binding only in the simultaneous presence of the RRM and GR regions. We proposed the participation of GR region in the recognition and binding of G4-RNA, thereby inducing the recruitment of RRM.

## Results

### ALS-linked mutations in the G4-RNA binding

**Figure 1:**
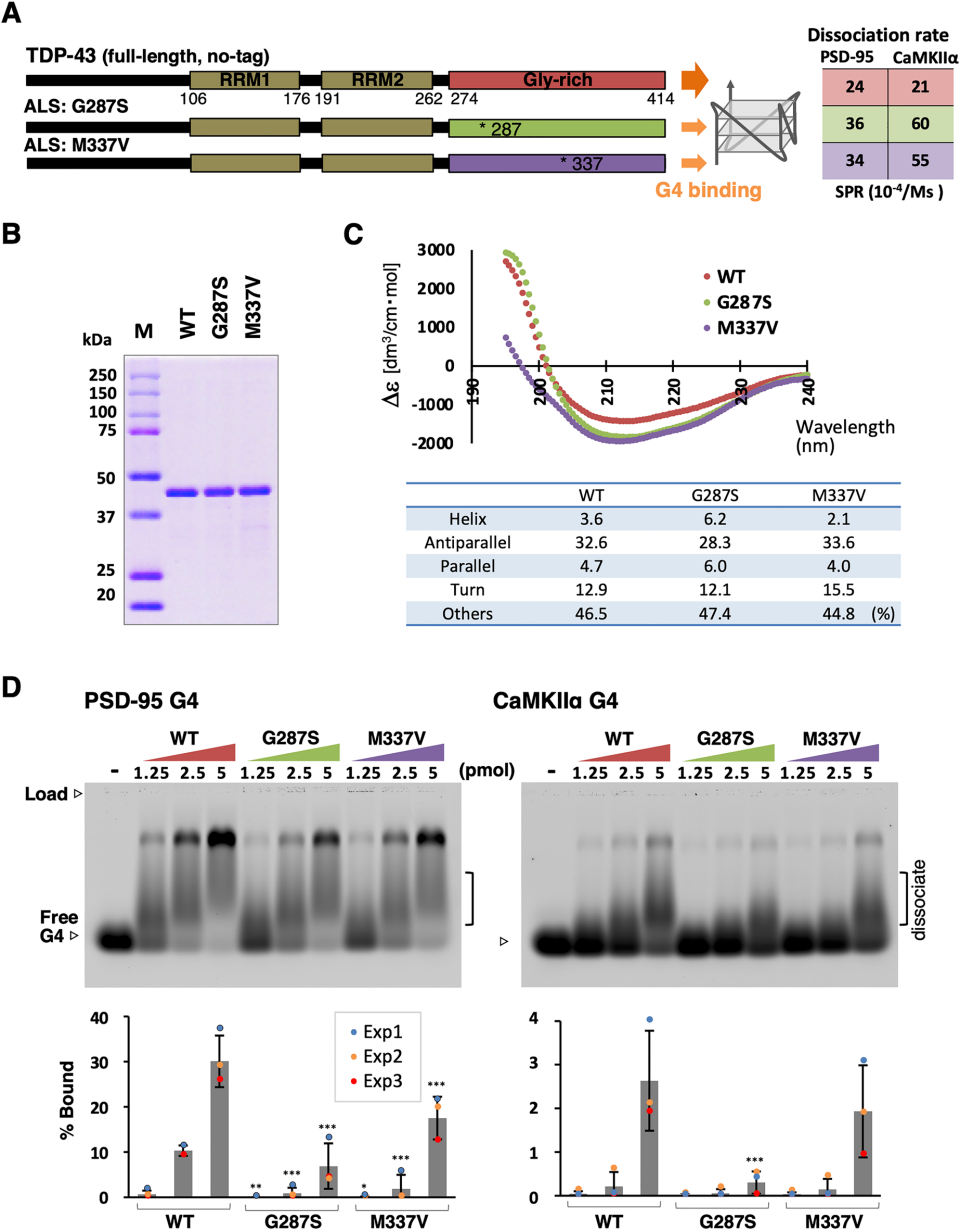
The ALS-linked mutations affect the interaction between human TDP-43 and G4-RNA. (**A**) The structural features of human TDP-43. The positions of two patient derived mutations are indicated. The dissociation rates of two types of G4 measured by SPR in previous studies are shown on the right^20^. (**B**) The purified untagged full-length dimeric proteins of wild-type and mutant proteins were used for this study. One microgram each of wild-type and mutant proteins were separated by 10% SDS-PAGE and detected by Coomassie Brilliant Blue staining (*for original images, see* Fig. S1A). (**C**) Far-UV CD spectrum of 0.2 μM of TDP-43 wild-type and two mutant proteins, TDP-43_G287S_ and TDP-43_M337V_. The x-axis represents wavelength, and the y-axis represents the five scans’ average molar CD (Δε). The percentages of specific secondary structure motifs calculated using the BeStSel algorithm are shown below (**D**) The agarose gel mobility shift assay. 0.5 pmol of fluorescently labeled G4 probes, PSD-95 and CaMKIIα, were mixed with the indicated amounts of TDP-43 wild-type and mutant proteins and electrophoresed under non-denaturing conditions. Each over-shifted band was quantified, and the standard error (± SEM) obtained after three independent experiments (Exp1, Exp2, and Exp3) is shown in the graph below. The statistical significance was determined by a two-tailed Student’s t-test *P < 0.05, **P < 0.01, ***P < 0.001. When electrophoresed under inappropriate pH conditions, most bound signals are detected at below as smear signals, indicating that these dissociated during migration (*see* Fig. S1B).

The recognition properties of TDP-43 have been analyzed in vitro using segment containing two RRMs^22–25^. However, the RRM of TDP-43 is not the only module that participates in RNA recognition^23,25^. Therefore, we purified a full-length untagged TDP-43 natural dimer and performed a more accurate RNA binding analysis^12,20,21^. Using PSD-95 (postsynaptic density protein 95) and CaMKIIα (calcium/calmodulin-dependent protein kinase II alpha subunit) (Table S2), two dendritic G4-mRNAs that bind TDP-43 *in vivo*^26,27^, we determined the effects of 10 ALS-linked amino acid substitution mutations^20^. All the mutant TDP-43 proteins showed reduced interaction with G4-RNA using SPR (surface plasmon resonance)^12,20^. In this study, we used these G4-RNAs and TDP-43 proteins that had the G287S or M337V mutations and the lowest G4-RNA binding properties (Fig. 1A).

First, to confirm the effects of ALS-linked mutations on the overall structure, far-UV circular dichroism (CD) spectroscopy was used (Fig. 1B,C). Secondary structure compositions of TDP-43 wild-type and mutant proteins were estimated using the BeStSel (Beta Structure Selection) algorithm (https://bestsel.elte.hu/index.php)^28^. TDP-43_G287S_ has a more helical content, whereas TDP-43_M337V_ showed a decrease and an increase in *β*-turn. We, therefore, hypothesized that the G287S and M337V mutations induced changes in the secondary and tertiary structures.

Next, to compare the interactions with G4-RNA under the unified experimental conditions of this study, an electrophoretic mobility shift assay was performed using a non-denaturing agarose gel (Fig. 1D). The binding of G4-RNA to specific interacting proteins with multiple binding modules often does not migrate normally when using a non-denaturing acrylamide gel^6^. As a result, this attempt was effective. By adding 2.5, 5, and 10 times the amount of protein to G4-RNA, we confirmed a dose-dependent over-shift in the G4-RNA mobility. The two mutant proteins showed a reduction in shifting bands, consistent with the SPR and secondary structure prediction data. The smeared bands of intermediate mobility might be dissociated RNAs during migration because the maximum signal is detected at this location when run at inappropriate pH condition for binding (Fig. S1B). This, combined with the recently reported results, we speculated that ALS-derived amino acid mutations alter the G4 associating structure of the GR region, resulting in the decreased direct binding affinity between G4-RNA and the GR region.

### ALS-linked mutations in GR segment do not affect direct binding to G4-RNA

**Figure 2:**
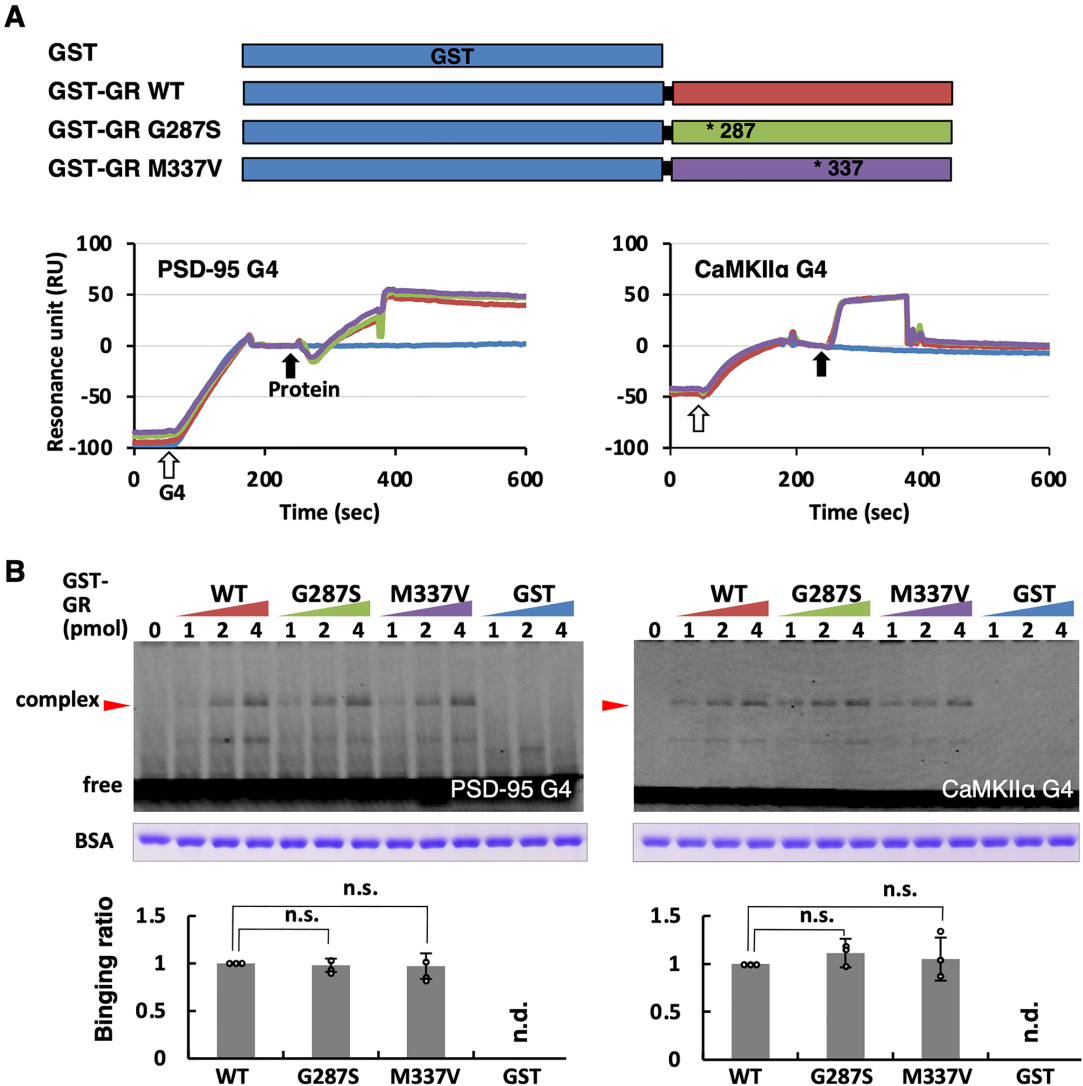
The ALS-mutations in the GR segment do not affect the binding to G4-RNA. (**A**) The structural features of GST fusion GR wild-type and mutant proteins and SPR sensorgrams of the interaction between G4-RNA and GST fusion proteins. Terminal biotinylated poly dT_16_ was bound to the streptavidin-coated sensor chip through streptavidin–biotin complex, to which poly-dA_16_ tailed G4-RNA (PSD-95 dA_16_ and CaMKIIα dA_16_, 20 nM) was immobilized by hybridization. The test protein (50 nM) was added to measure interaction with G4-RNAs. (**B**) UV cross-linking for detection of G4-RNA and binding protein complexes. The reaction mixtures were irradiated at 254 nm at 200 mJ/cm^2^ and analyzed by denaturing gel mobility shift assay. Shifting bands were enhanced in both fluorescent probes (Table S2) with increased GST-GR proteins (1, 2 or, 4 pmol each). Several minor bands are seen that may have been cleaved during electrophoresis, but only the major band was used for quantification (*for original images, see* Fig. S2). The gel was stained with Coomassie Brilliant Blue and the blocking agent BSA signals were used to correct the data as a loading control. The experiments were performed thrice, and the y-axis values represent the mean ± SEM at 4 pmol. The statistical significance was not confirmed by a two-tailed Student’s t-test (n.s.).

We confirmed the binding affinity to G4 by a segment of the GR region alone fused to GST (glutathione S-transferase)^20^. To confirm the effects of the mutations, we examined the possible influence of GST-fused GR mutant proteins on the G4-RNA immobilizing sensor chip using SPR (Fig. 2A). In the case of GST-GR fusion, however, no significant difference was observed in the interaction with G4-RNA between the wild-type GST-GR and mutant GST-GRs. To confirm this contradictory result between the full-length TDP-43 and the GR segment, these interactions were further analyzed by gel shift assay. No shifting bands were detectable on agarose gels, suggesting that additional binding modules were required for the GR region to form a stable complex (Fig. S1C). It was possible that the GR region alone cannot withstand even slight structural changes in gels forced to operate under low-salt conditions^29–31^. Therefore, we reinforced the association by UV cross-linking and confirmed the complex by SDS-PAGE^21^. The cross-linking and gel shift assay supported the SPR results, and two mutations did not affect the interaction of GST-GR with G4-RNAs (Fig. 2B). We repeated the experiment with different binding conditions such as buffer and temperature; however, the results were consistently reproducible.

These unexpected results suggested that the mutations in the GR region do not affect GR-G4-RNA binding but influence other molecular characteristics of GR such as the modulation of G4-RNA structures and/or intramolecular signal transmission to the RRM for modulation of its RNA-binding activity. Indeed, mutations or deletions of G4 binding proteins have been reported to alter the G4 conformation^32,33^. Therefore, we analyzed the conformational changes in G4-RNA caused by disease-related amino acid substitutions in the GR region.

### Alteration of G4-RNA structure after binding to GR mutant proteins

**Figure 3:**
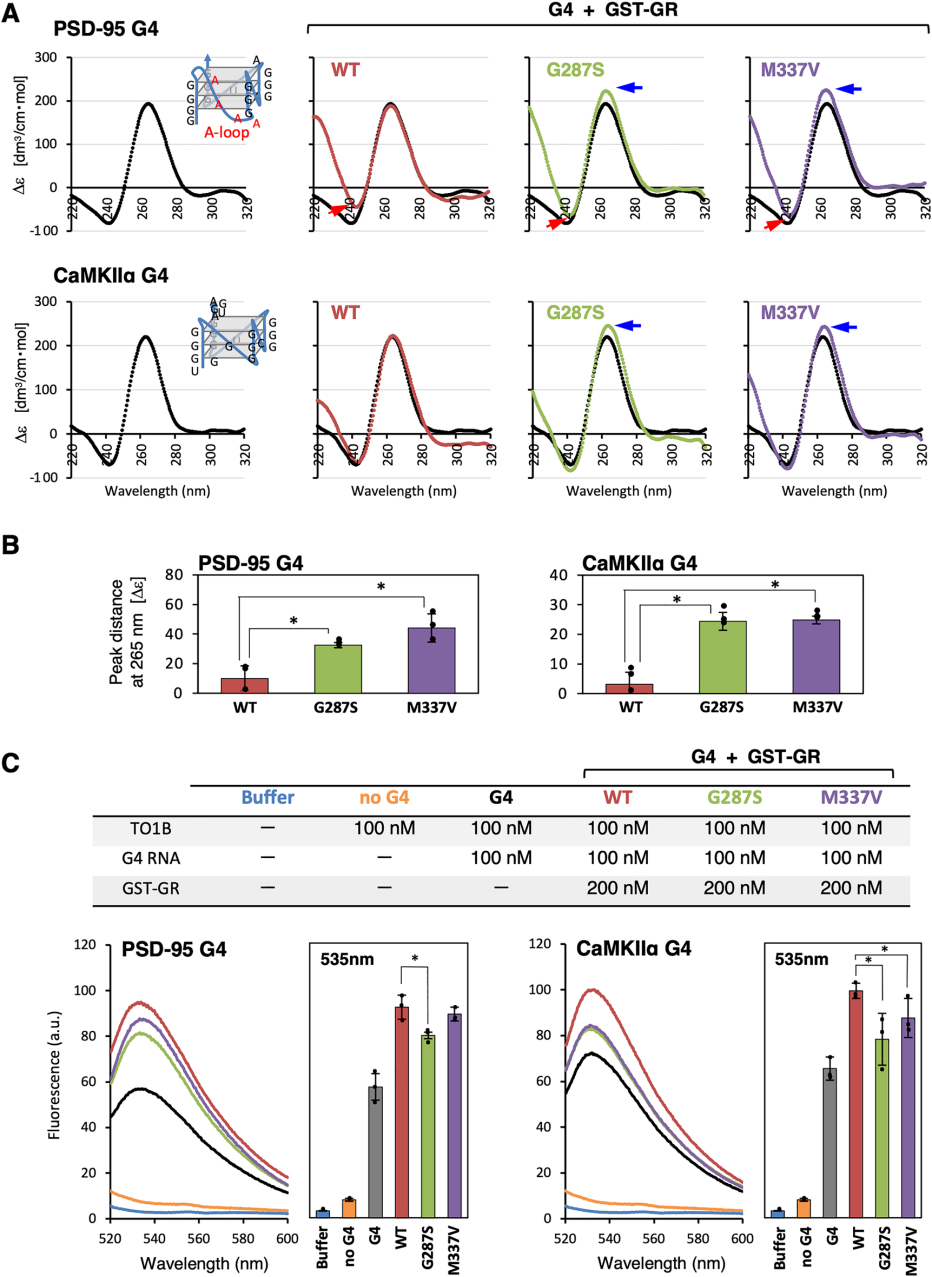
The GR mutant proteins alter the G4 structure. (**A**) CD spectra of G4-RNAs, PSD-95 and CaMKIIα (Table S2) in the absence and presence of wild-type and mutant GST-fused GR proteins. The blue arrows indicated that positive peaks were due to the conformational effects of G4-RNAs. The red arrows indicated that negative peaks were due to the structure of the poly-A loop and were affected by protein binding. CaMKIIα without poly-A did not show this decrease in negative peaks. The x-axis represents wavelength, and the y-axis represents the five scans’ average molar CD (Δε). The CD spectrum values of G4-RNA plus GST-fused GR protein were subtracted from each protein spectrum (*For protein spectra, see* Fig. S3). (**B**) The change of the positive peak at 265 nm by the addition of each GST-GR proteins from (A). The experiments were performed thrice, and the y-axis values represents the mean ± SEM. The statistical significance was determined by a two-tailed Student’s t-test *P < 0.05. (**C**) The measurement of G4 levels using the G4-binding fluorescent sensor TO1B. The fluorescence emission spectra of TO1B after binding with two G4-RNAs (PSD-95 and CaMKIIα) were measured in the absence or presence of GST-GR wild-type or mutant proteins. The buffer control and G4-independent fluorescence intensity are also shown. The graphs on the right showed the average fluorescence intensities at 535 nm. The experiments were performed thrice, and the y-axis values represents the mean ± SEM. A two-tailed Student’s t-test determinedthe statistical significance *P < 0.05. The effects of the three proteins on G4-independent fluorescence intensity are shown in Fig. S4.

The possible impact of GR mutations on the conformations of G4-RNA, PSD-95 and CaMKIIα was analyzed by CD spectroscopy, which is frequently used to evaluate the conformational properties of nucleic acids. Recently, we identified that the CD spectral patterns of these two G4-RNAs were essentially the same even after binding of the full-length wild-type TDP-43, unlike FUS (fused in sarcoma)^20^. TDP-43 binds to cover the entire G4-RNA and maintains a stable conformation^20^. A positive peak near 265 nm and a negative peak near 240 nm characterized the parallel-stranded G4 conformation (Fig. 3A). Wild-type GST-fused wild-type GR segment, GST-GR_WT_, did not affect the positive peak of the CD spectral pattern, as expected. However, the presence of the mutant proteins, GST-GR_G287S_ or GST-GR_M337V_, both increased the positive peak due to conformational alternation of G4-RNAs (Figs. 3A,B and S3), which is generally characterized as an altered stacking arrangement of guanine tetrads^15,34^. In addition, binding of the mutant proteins affected the negative peak of PSD-95 G4-RNA. This observation supported the prediction that the structure of the poly-A loop was affected by the binding^35^.

Next, the effects on the G4 stabilization were analyzed by a turn-on assay using biotinylated thiazole orange, TO1B. Although, the biotin modification significantly reduces non-specific interactions with RNA, G4 structures can rigidify TO1B and enhance the fluorescence^36–38^. The wild-type GST-GR protein increased G4-dependent fluorescence enhancement, but the ALS-linked mutations G287S and M337V inhibited the increase (Fig. 3C). In the absence of G4, no enhancement of TO1B fluorescence was observed by adding any protein (Fig. S4).

From the CD spectroscopy and the TO1B turn-on assay together, we concluded that mutations altered the maintenance of the G4-RNA conformation. Does this GR mutation-induced structural change in G4 affect other RNA-binding modules such as RRM, altering its overall affinity for G4-RNA? The isolated RRMs have limited individual affinity for RNA, but the participation of partner regions with RNA recognition complements stable binding^39^. Full-length-TDP-43 specifically recognizes only parallel-stranded G4, whereas the RRM segment alone exhibits broad binding affinities with DNA/RNA including UG-rich sequences^12,20,40,41^.

### RRM recognizes G4-RNA

**Figure 4:**
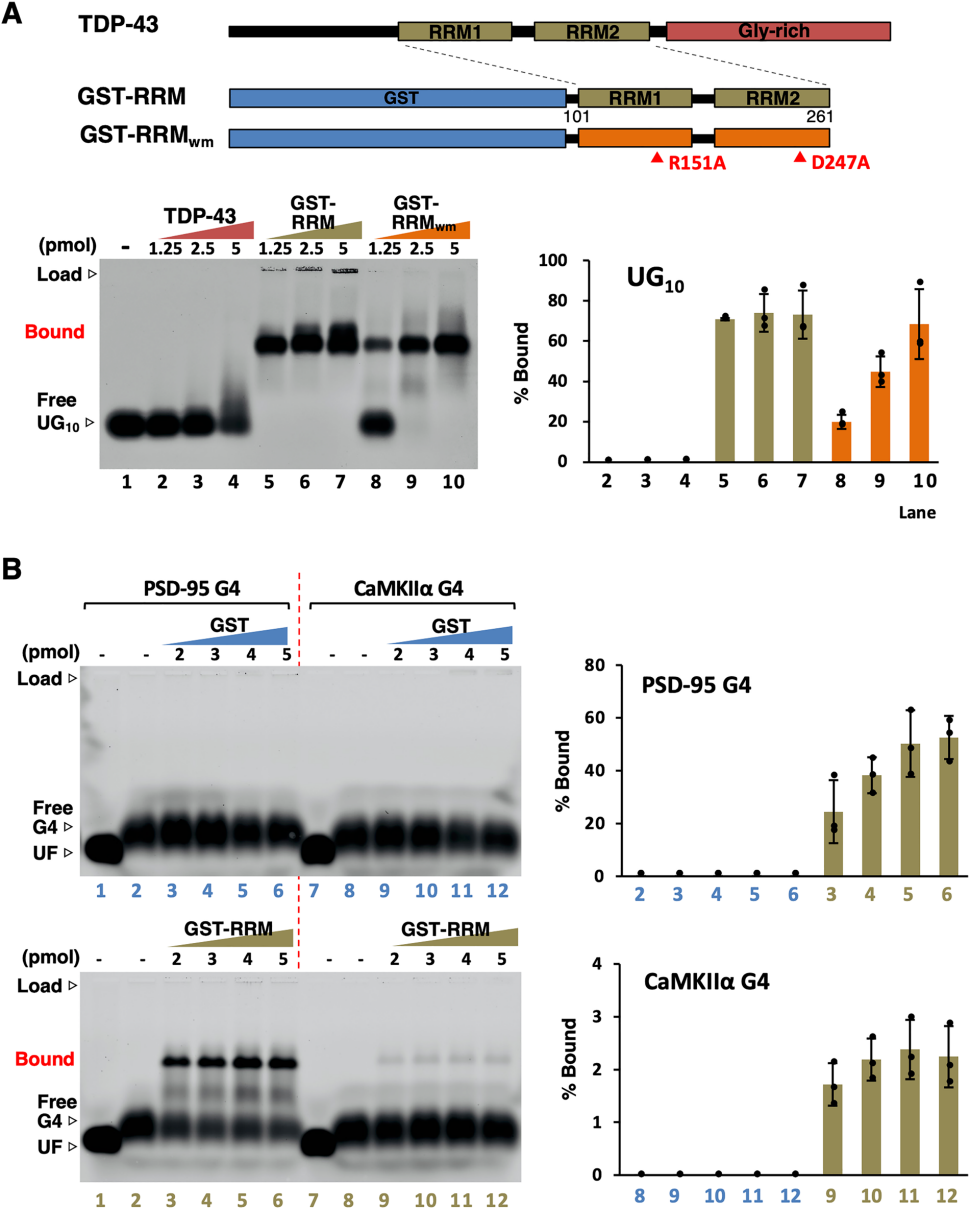
The binding specificity of RRM. (**A**) The structural feature of GST-fused RRM and double-mutant protein, RRM_wm_ are indicated. To confirm the reproducibility under the experimental conditions different from the previous study, the binding to the fluorescently labeled UG_10_ probe (0.5 pmol) was confirmed by agarose gel mobility shift assay using an increased amount of TDP-43, GST-fused RRM, and RRM_wm_. (**B**) Agarose gel mobility shift assay using two G4 probes (0.5 pmol) and increased amount of GST-fused RRM. No band shift was observed in the GST-only control, but a concentration-dependent shift was confirmed with GST-RRM. Lanes 1 and 7 were run simultaneously with unfolded RNA heat denatured with 50% formamide. The experiments were performed thrice, and the y-axis values represent the mean ± SEM.

RRM is one of the most abundant RNA-binding modules^42,43^. The isolated RRM binds the UG-rich sequence *in vitro*^22,24,44,45^, whereas in vivo screening of the full-length TDP-43 interacting RNAs revealed that UG-rich sequences were neither necessary nor sufficient to specify a TDP-43 binding site^27^. Our previous study obtained by SPR analysis supported these findings and showed that, unlike the readily binding RRM segment, full-length native homodimer has no affinity for UG-rich sequence and binds only parallel-stranded G4 structures^12,20^. However, it remains unclear whether isolated RRM interacts directly with the G4 motif. Therefore, we performed a gel mobility shift assay to confirm the reproducibility of protein-UG-rich interaction under the same experimental conditions employed in this study (Figs. 1D and 4A). As reported in a previous study^12^, no binding was detected even when UG_10_ probe was reacted with 10 times the amount of full-length TDP-43 dimer (Table S2). On the other hand, the isolated RRM segment over-shifted most of the signals even at the lowest amount. The double-mutant RRM segment, which harbors two amino acid substitutions deleterious to RNA binding, GST-RRM_wm_ (R151A and D247A within RRM1 and RRM2)^24^, showed conditional reduction of binding at low concentrations, but when 10 times the amount was added, a binding signal almost equivalent to that of the wild-type RRM segment was detected. The results obtained corroborated our previous report^12^; therefore, we analyzed the interaction between RRM and G4-RNA.

RRM could confirm the band shift of both PSD-95 G4 and CaMKIIα G4, but there was a difference in the binding affinity, *i.e.*, higher for PSD-95 G4 harboring A-loop (Fig. 4B). While this trend was similar for the full-length dimeric protein (Fig. 1D), there was no significant difference between PSD-95 G4 and CaMKIIα G4 for the GR protein cross-linked with G4-RNA (Fig. 2B). This indicated that the strength of TDP-43 binding to G4-RNA is derived from its interacting affinity to RRM rather than the GR region. At the same time, it was suggested that G4 recognition by RRM is hypersensitive to conformational changes of G4.

### Mutations in the GR region interfere with the recruitment of RRM to G4-RNA

**Figure 5:**
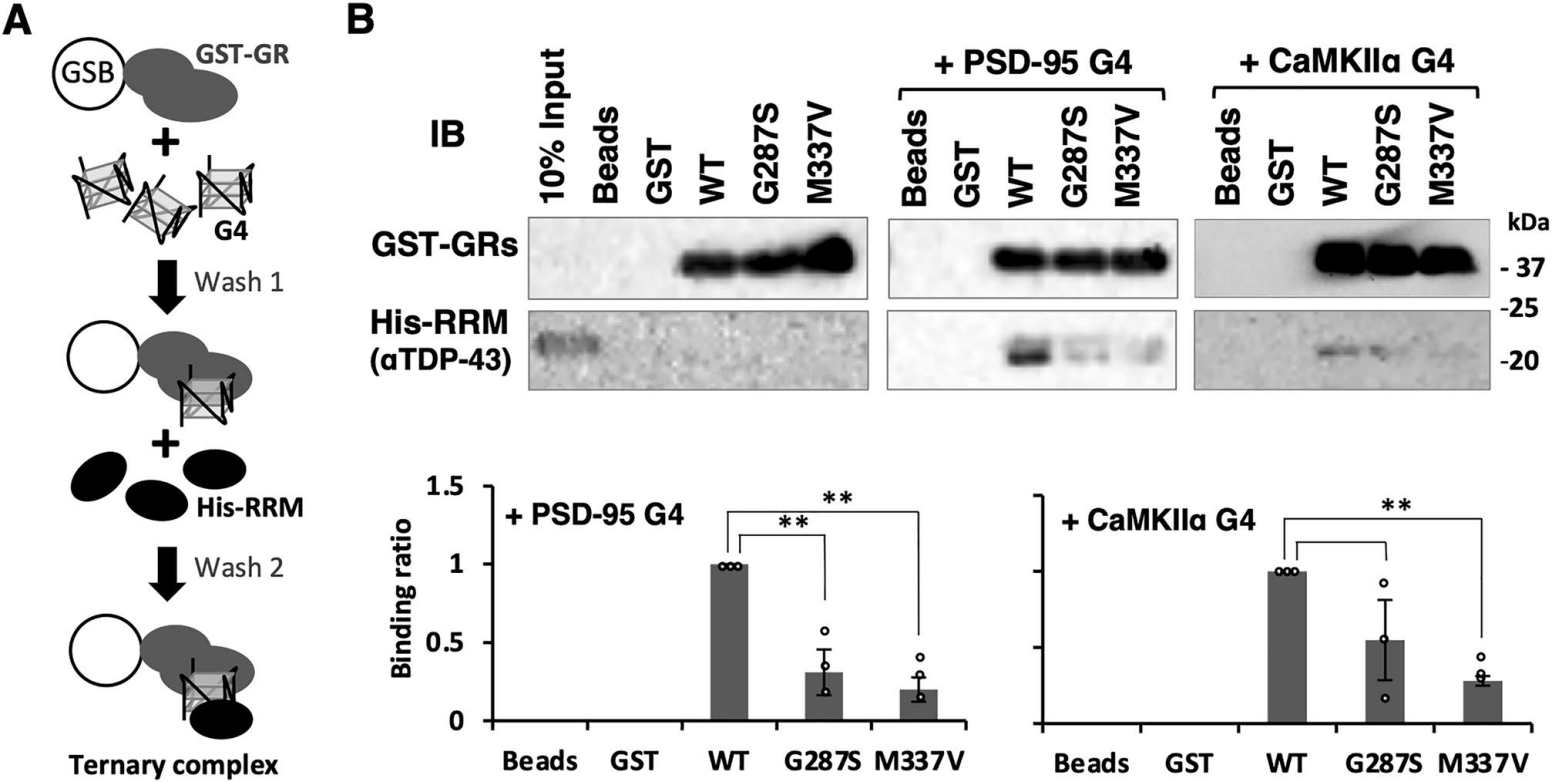
GR mutations interfere with the recruitment of RRM. (**A**) A cartoon of experimental flow. (**B**) The His-RRM protein (Fig. S5) was co-pulled with GST-GR wild-type and mutant proteins by glutathione sepharose beads (GSB) in the absence and presence of G4-RNAs, PSD-95 and CaMKIIα (Table S2) and detected by western blotting analysis (*for original images, see* Fig. S6). The graphs show the average of G4 dependent binding ratio compared to the GST-GR wild-type protein and the SEM of three experiments is shown. Two-tailed Student’s t-test was used to determine the statistical significance **P < 0.01.

Since both GR region and RRM bind to G4-RNA, they may cooperate in a specific recognition and stable binding. To examine this hypothesis, we prepared a His-tagged RRM segment and examined its binding activity to GST-GR in the presence and absence of G4-RNA (Figs. 5A and S5). First, G4-RNA was bound to GST-GRs immobilizing beads, and after washing, His-tagged RRM detected the ternary complex formation. The bound RRM was then detected by western blotting (Fig. 5B). Co-precipitation of the wild-type GST-GR with His-tagged RRM was observed in the presence of G4-RNAs (PSD-95 and CaMKIIα). In contrast, two GR mutant proteins, GST-GR_G287S_ and GST-GR_M337V_, significantly reduced the levels of His-RRM precipitation. Under this condition, we could not detect any G4-RNA-independent interaction between the RRM and GR regions. This finding indicated the G4 structure dependent intramolecular organization of RRM and GR regions, leading to modulation of its G4-RNA binding property.

## Discussion

Although TDP-43, the major protein responsible for ALS, is an RNA-binding protein, its binding target of complete dimer form has remained unknown for a long time. Based on a SELEX (Systematic Evolution of Ligands by Exponential Enrichment) screening, we first identified a parallel-stranded G4 structure as a specific binding target, and proposed G4-containing RNAs as the initial selection signal of mRNAs to be transported into neurites for local translation by TDP-43^12,15,20^. In this mRNA selection process, the GR region is essential. Moreover, the RRM of TDP-43 is a rather promiscuous binding module with a high affinity for RNA. When exposed alone, RRM prefers a UG-repeat sequence for which full-length native TDP-43 has no affinity^22,24,35^.

We hypothesized that G4-RNA binding affinity exists independently on the RRM and GR regions, and ALS-linked amino acid substitutions in the GR region of TDP-43 simply resulted in reduced binding of the GR region. Unexpectedly, the GR region with the two mutations herein analyzed showed the same G4-RNA binding ability as the wild-type. Hence, it is interesting that the CD spectra and TO1B turn-on assay showed the two amino acid mutations affected the G4 conformation. A subsequent ternary complex formation experiment suggested that the conformational change of G4-RNA interferes with the recruitment of RRM and reduces the binding affinity of TDP-43.

Regarding binding specificity, the essential fact is that the RNA-binding module, the RRM segment, bind the UG-rich sequences, whereas the full-length TDP-43 completely loses this binding capacity. We interpreted that the loss of strong RNA binding affinity may be the result of a perturbation by another intramolecular module such as the GR region, thereby inhibiting the binding to targets other than parallel-stranded G4-RNA. Another possibility is the involvement of the N-terminal domain (NTD). It has been reported that NTD behaves as a scaffold for nucleic acid binding and contributes to binding specificity^23,25^. Furthermore, a compound that targeting NTD inhibits RRM-RNA binding via allosteric modulation^42^. However, we have so far failed to detect inhibition of the binding between RRM and UG-rich RNA by an isolated GR region or NTD.

Over 50 missense mutations have been identified in the TDP-43 cording gene in familial and sporadic patients^46^. Most of these amino acid substitutions were in the GR region. It remains unclear whether all of them interfere with RRM recruitment via the conformational changes in the G4-RNA structure without affecting G4 binding, as determined in this study. Further detailed analysis will be carried out to determine whether there are amino acid substitutions that affect direct interaction with G4. In addition, we recently found that G4-RNA promotes LLPS during FUS RNP granule formation^47^. The GR region of TDP-43 was involved in LLPS^48,49^, and interaction with G4-RNA may have complex effects. The insights provide essential information for the studying G4-binding proteins involved in ALS pathogenesis.

## Materials and methods

### Plasmids

*E. coli* expression plasmid pGEX-GR_WT_ was described previously^20^. pGEX-GR_G287S_ and pGEX-GR_M337V_ were constructed by PCR-based mutagenesis method using several primer pairs^20^. *E. coli* expression plasmid pET-His-RRM was constructed by replacing the cDNA fragment between the EcoRI and BamHI sites of pGEX-RRM (101-261aa) between the same sites of pET28a ( +) (Merck, NJ USA)^12^.

### Proteins

Full-length, untagged natural dimer proteins TDP-43_WT_, TDP-43_G287S_ and TDP-43_M337V_ were described previously^12,20^. Glutathione S-transferase (GST) and GST fusion proteins, GST-GR_WT_, GST-GR_G287S_ and GST-GR_M337V_ were purified as described^20^. His-RRM was overproduced in *E. coli* BL21(DE3) carrying a pET-His-RRM plasmid as noted above. The transformants were grown at 30 °C up to OD_600_ = 0.4, and then Isopropyl β-D-1-thiogalactopyranoside (IPTG) was added at a final concentration of 0.5 mM. After 4 h of culture at 30 °C, the cells were harvested by centrifugation and suspended in lysis buffer (10% glycerol, 50 mM Tris–HCl pH 8.0, 250 mM NaCl, 10 mM 2-mercaptoethanol, 0.1% Nonidet-P40, 10 mM imidazole and 0.1% benzamidine hydrochloride) containing 1.5 mg/ml lysozyme, stored for 30 min on ice, and then sonicated. After centrifugation, the supernatant was loaded onto the Ni–NTA agarose (Qiagen, Hulsterweg Nederland) open column. The column was washed with lysis buffer and the bound protein was eluted with a buffer containing 250 mM imidazole.

All proteins were further purified by Superdex 75 10/300 GL (Sytiva, MA USA) (10% glycerol, 20 mM HEPES [4-(2-hydroxyethyl)-1-piperazineethanesulfonic acid]-NaOH, pH 6.8, 300 mM NaCl and 0.1 mM EDTA) and the peak fractions were collected with use of ÄKTA explorer 10 S/100 system (Sytiva, MA USA). The purified proteins were concentrated by a centrifugal filter (Vivaspin 6-10 kDa; Sytiva, MA USA) down to 0.5 mg/ml and stored frozen at −80 °C.

### Agarose gel mobility shift assay

The fluorescence-labeled oligonucleotides, Cy3-PSD-Ax_647_, Cy3-CaMKII-Ax_647_ and UG10- Ax_647_ (Table S2) (0.5 pmol) were incubated with the indicated amount of diluted protein in a 10 μl buffer (10% glycerol, 20 mM PIPES–NaOH pH 6.8, 0.8 mM MgCl_2_ and 150 mM NaCl) containing 0.1 mg/ml bovine serum albumin (BSA) and 0.1 mg/ml poly-dI-dC at 25 °C for 30 min. The samples were loaded onto a 1% agarose gel run at 50 V for 60 min at 25 °C. Although the buffer was 0.5 × TBE (pH 6.8), only the dissociation assay was performed under the condition of pH 8.3 (Fig. S1B). The gels were visualized and quantified by Typhoon 9410 imaging system (Sytiva, MA USA). The unfolded RNA samples were prepared by heat denaturation with 50% formamide at 98 °C for 3 min prior to agarose gel electrophoresis, indicated as “UF” in the diagram.

### Surface plasmon resonance (SPR) analysis

SPR analysis was carried out as described previously^20^ in the SPR buffers (20 mM PIPES [1,4-Piperazinediethanesulfonic acid sesquisodium salt]-NaOH, pH 6.8, at 25 °C, 12 mM KCl, 0.8 mM MgCl_2_, 0.05% Tween-20, and 150 mM NaCl) using a BIAcoreJ instrument (Sytiva, MA USA) at 25 °C. In this method, simultaneous analysis was performed using two flow cells. The dT_16_ oligomer was immobilized onto flow cell 2 and flow cell 1 was left blank to serve as an in-line reference surface. The analyte protein was injected into the flow cells-1 and -2 of the sensor chip. The plasmon resonance values (resonance unit; RU) were obtained from the flow cell-2 data after subtracting the flow cell-1 data.

### Circular dichroism (CD) spectra analysis

For conformational analysis of the wild-type and mutant TDP-43 proteins (0.5 mg/ml), the purified protein samples were diluted in PBS (phosphate-buffered saline) to make a 0.2 μM solution, and then subjected to CD analysis with by using a spectropolarimeter (J-820; Jasco, Tokyo Japan)^47^. The estimated G4 structure of the synthesized RNAs, PSD-95 and CaMKIIα (Table S2) was confirmed by CD spectra analysis using 1 μM oligonucleotide in each of the SPR buffer conditions at 25 °C by using a spectropolarimeter^21^. To analyze the influence of protein binding on G4 conformation, parallel-stranded G4-forming RNAs, PSD-95 and CaMKIIα, were confirmed by CD spectra analysis using 1 μM RNA in the presence or absence of 1 μM protein in the CD buffer (20 mM PIPES–NaOH pH 6.8, 0.8 mM MgCl_2_ and 150 mM NaCl) for 30 min at 25 °C. The scan was repeated five times, and Jasco Spectro Manager compiled the mean values. The x-axis represents the wavelength, whereas the y-axis represents the molar CD (Δε). The content of secondary structure was estimated by using the BeStSel online deconvolution webserver^28^.

### UV cross-linking and gel shift assay

The fluorescence-labeled oligonucleotides, Cy3-PSD-Ax_647_ and Cy3-CaMKII-Ax_647_ (Table S2) (0.5 pmol) were incubated with the diluted protein (1, 2, or 4 pmol each) in a 10 μl buffer (10% glycerol, 20 mM PIPES–NaOH pH 6.8, 0.8 mM MgCl_2_ and 150 mM NaCl) containing 0.1 mg/ml BSA. The mixtures of G4-RNAs and proteins were subjected to cross-linking upon exposure to 254 nm UV at 200 mJ/cm^2^ using a UV cross-linker, FS-1500 (Funakoshi, Tokyo Japan). The cross-linked samples were separated by 10% SDS-PAGE and the fluorescent signal (Ax_647_, Alexa Fluor 647) was scanned by Typhoon 9410 imaging system (Sytiva, MA USA)^21^.

### TO1B turn-on assay

The oligonucleotide (20 pmol) was incubated with the protein (40 pmol) in 180 μl buffer (20 mM PIPES-NaOH, pH 6.8, at 25 °C, 0.8 mM MgCl_2_, 0.05% Tween-20, and 150 mM NaCl) for 10 min at 25 °C. Twenty microliters of 1 μM thiazole orange-3PEG-Biotin, TO1B (ABM, BC Canada) was mixed in the same buffer and stored for 5 min at 25 °C. After 5 min, the ligand-enhanced fluorescence spectra were obtained using a fluorescence spectrometer (Shimadzu RF-5300PC) with an excitation wavelength of 505 nm and a scanning emission wavelength of 520–600 nm. The fluorescence intensities at 535 nm were used to plot the comparison graphs. The oligonucleotides used in this study were obtained from GeneDesign, Inc. (Osaka Japan) (Table S2).

### GST pull-down assay

Glutathione sepharose beads (5 μl) (Sytiva, MA USA) immobilized with 4 μg of GST or GST fusion protein were incubated with G4-RNA, PSD-95 or CaMKIIα (Table S2) (800 pmol) in 250 μl of binding buffer at 4 °C for 2 h in binding buffer a 250 μl (10% glycerol, 20 mM PIPES-NaOH pH 6.8, 0.8 mM MgCl_2_ 1.8 mM CaCl_2_ and 145 mM NaCl, 5.4 mM KCl and 0.05% Tween-20) containing 0.1 mg/ml BSA. These beads were washed two times and incubated with two μg of His-RRM in a 300 μl binding buffer containing 0.1 mg/ml BSA at 4° C for 2 h. These beads were washed thrice, and interacting proteins were eluted with 12.5 μl of 1 × SDS sample buffer. The samples were separated by 12.5% SDS**-**PAGE, transferred onto a porvinilidendifluoride membrane (Immobilon-P, Merck, NJ USA), and anti-TDP-43 antibodies (Cell Signaling Technology, MA USA) were used for detection as described previously^20^.

## Supplementary Information

Supplementary Information.

## Data Availability

The data sets generated during and/or analyzed during the current study are available from the corresponding authors on reasonable request. (akira.ishiguro.iu@hosei.ac.jp).
