# Supplementary material for: ALS-linked TDP-43 mutations interfere with the recruitment of RNA recognition motifs to G-quadruplex RNA
Source: Sci Rep. 2023 Apr 12;13:5982. doi: 10.1038/s41598-023-33172-5 (PMC10097714; doi:10.1038/s41598-023-33172-5)
Supplement: Supplementary file 1 — Supplementary Information. [file 41598_2023_33172_MOESM1_ESM.pdf]

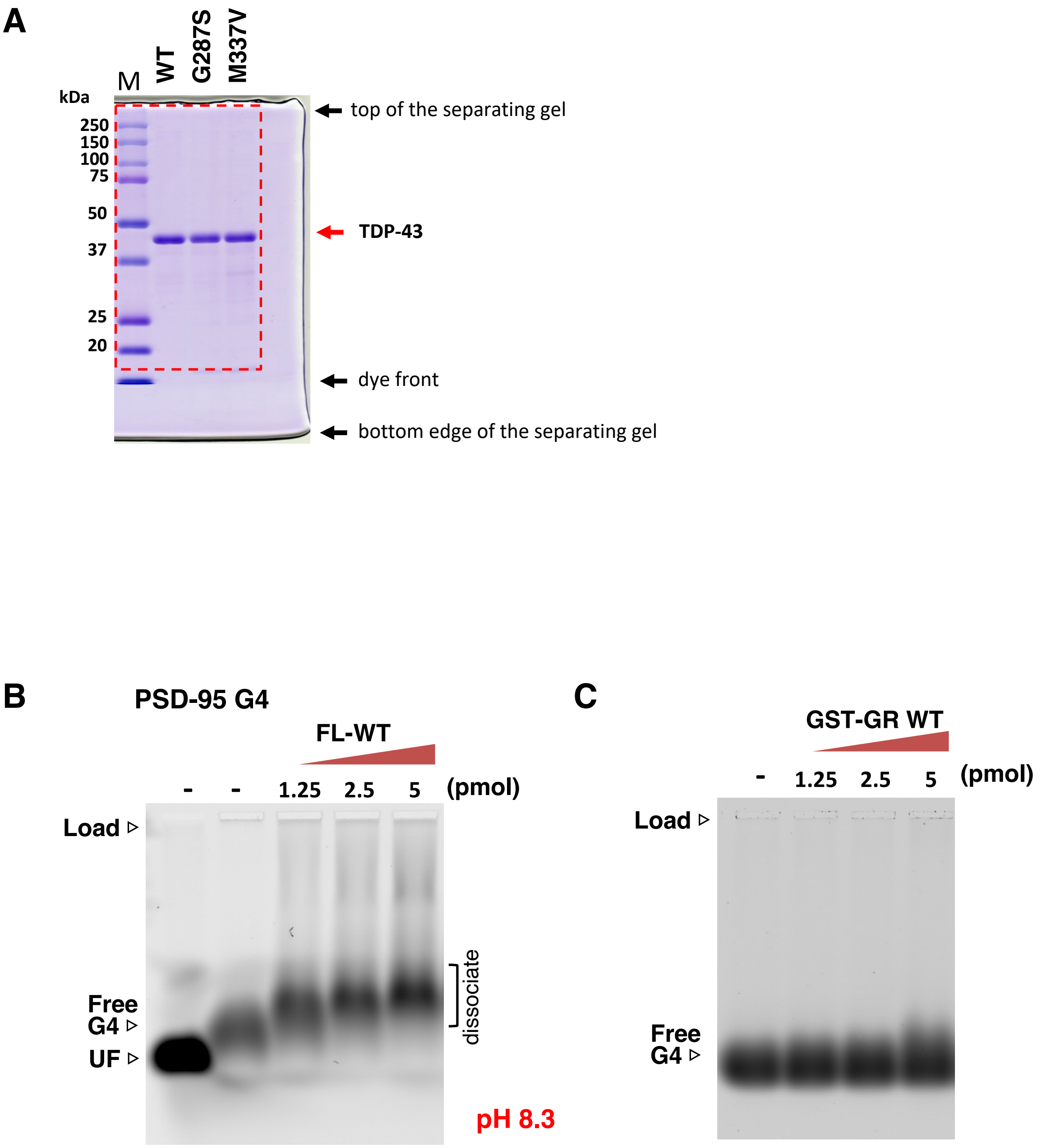

Supplementary Figure S1. Agarose gel mobility shift assay. [Related to Fig. 1 and Fig. 2]. (**A**) The purified wild-type and mutant proteins. Areas enclosed by red dashed lines are used in Fig. 1B. (**B**) Electrophoresis conditions, pH 8.3 that deviate significantly from the optimal pH for TDP-43 and G4 interaction cause most of them to be dissociated during electrophoresis. The leftmost lane is the sample after heat denaturation with formamide (UF). (**C**) The interaction between the GST-fused GR fragment protein and G4 could not be confirmed by agarose gel mobility shift assay.

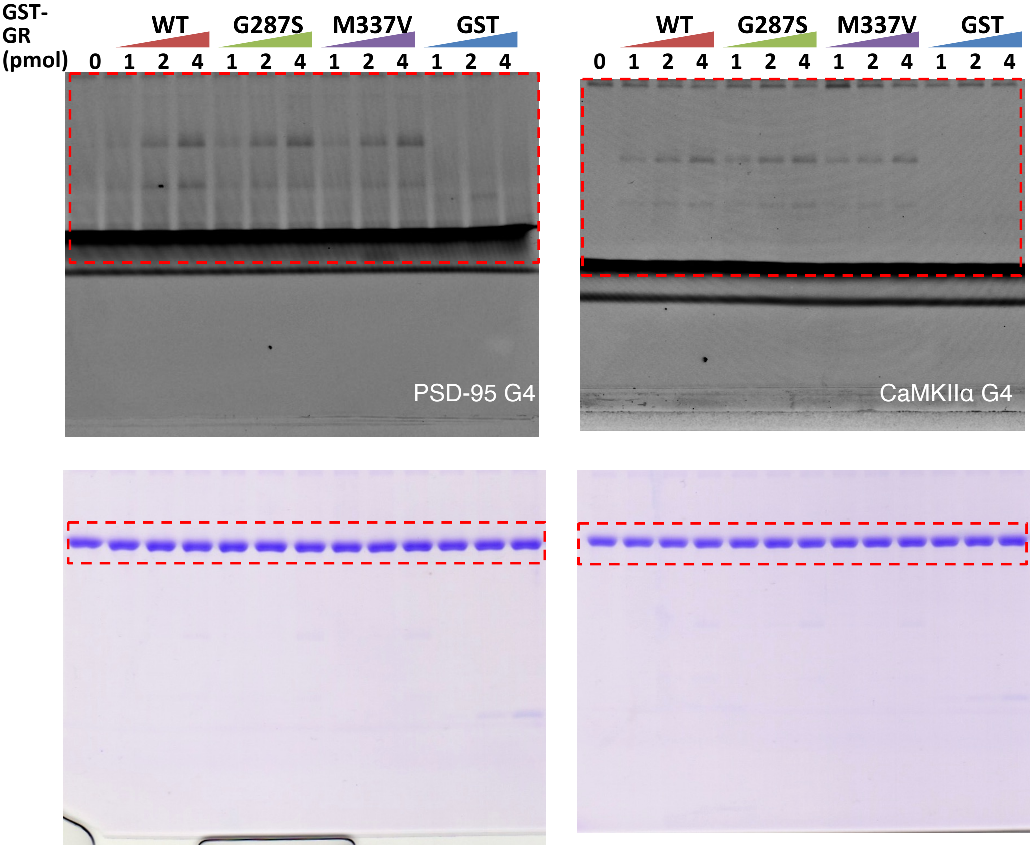

Supplementary Figure S2. Original images of gel shift assay. [Related to Fig. 2B] Areas enclosed by red dashed lines are used in Fig. 2B.

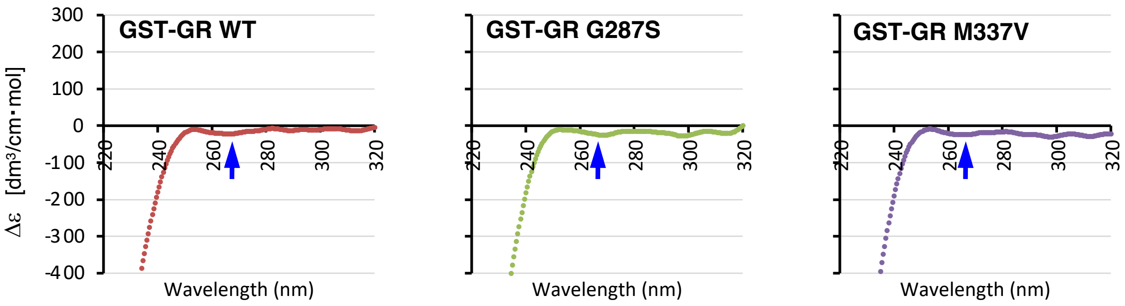

Supplementary Figure S3. CD spectral patterns without RNA. [Related to Fig. 3A] CD spectra of GST fusion proteins, wild-type and mutant proteins, G287S and M337V in the absence of G4-RNA. Blue arrows indicate the positive peak positions of G4 RNAs.

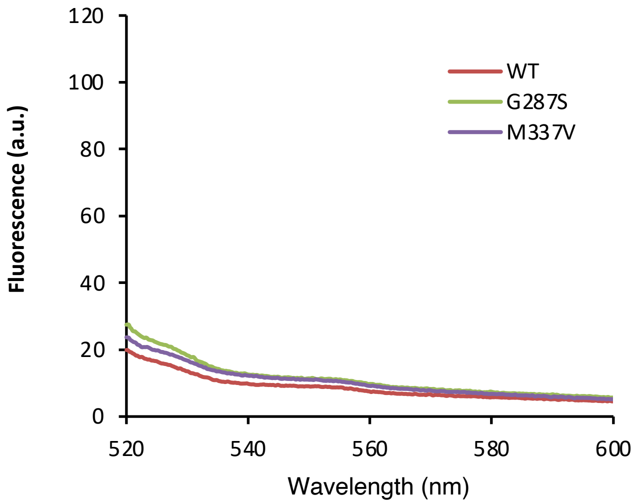

Supplementary Figure S4. [Related to Fig. 3C] The fluorescence intensity without G4-RNA**.** The fluorescence intensity of TO1B (100 nM) after adding GST-GR wild-type or mutant proteins (200nM) are shown as controls.

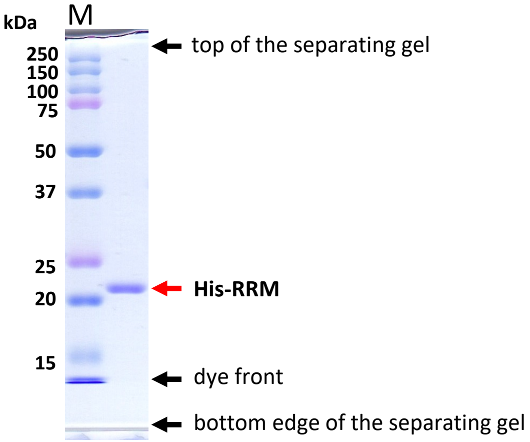

Supplementary Figure S5. Purified His-RRM used for this study. [Related to Fig. 5] His-RRM protein one µg was separated by 12.5 % SDS-PAGE and detected by staining with Coomassie Brilliant Blue.

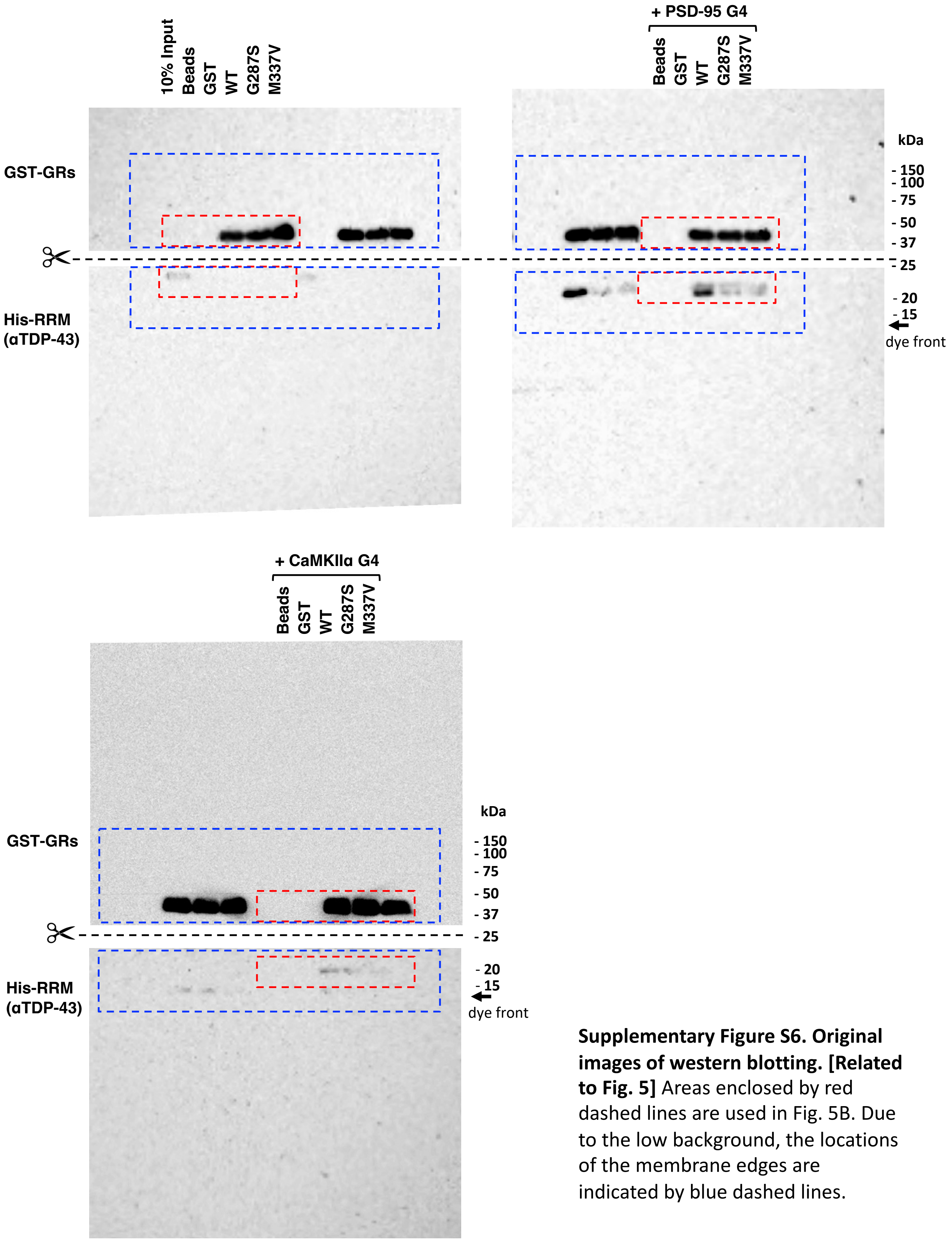

Supplementary Figure S6. Original images of western blotting. [Related to Fig. 5] Areas enclosed by red dashed lines are used in Fig. 5B. Due to the low background, the locations of the membrane edges are indicated by blue dashed lines.

Supplementary Table S1.
ALS-associated/ potentially related RNA binding proteins and RNAs.
Gene products for which mutations have been found in patients (1-15), and potentially related (16-19) are shown.

	RBP / RNA	Molecular characterization	Related biomolecular assembly
1.	**SETX (ALS4)**	resolves RNA-DNA hybrids, and controls G4 DNA (Salvi & Mekhail, 2015)	RNP granules (Marmor-Kollet *et al.*, 2020)
2.	**Zfp106 (ALS5)**	interacts with G_4C_2 repeats that can form G4 (Celona *et al.*, 2017)	nuclear puncta (Celona *et al.*, 2017)
3.	**FUS / TLS (ALS6)**	G4 binding protein (Oyoshi & Kurokawa 2012; Imperatore *et al.*, 2020)	RNP granules (Fujii *et al.*, 2005; Bosco *et al.*, 2010; Ishiguro *et al.*, 2021)
4.	**ANG (ALS9)**	mediates stress-induced tiRNAs assume G4 structures (Ivanov *et al.*, 2014)	RNP granules (Emara et al., 2010)
5.	**TDP-43 (ALS10)**	G4 binding protein (Ishiguro *et al.*, 2016; Ishiguro *et al.*, 2021a)	RNP granules (Molliex *et al.* 2015; Conicella *et al.*, 2016; Schmidt & Rohatgi, 2016)
6.	**ATXN2 (ALS13)**	suppresses the formation of RNA-DNA hybrids, and preventing DNA G4 formation (Salvi & Mekhail, 2015)	RNP granules (Nonhoff et al., 2007; Sudhakaran *et al.*, 2014)
7.	**hnRNPA1**	G4 binding protein (Zamili *et al.*, 2014; Liu & Xu, 2018)	RNP granules (Guil *et al.*, 2006)
8.	**hnRNPA3**	G4 binding protein (Mori *et al.*, 2013; von Hacht *et al.*, 2014)	RNP granules (Mori *et al.*, 2016)
9.	**hnRNPA2/B1**	G4 binding protein (Scalabrin *et al.*, 2017; von Hacht et al., 2014)	RNP granules (Martinez *et al.*, 2016)
10.	**EWSR1 / EWS**	G4 binding protein (Takahama *et al.*, 2011)	RNP granules (Andersson *et al.*, 2008)
11.	**TIA1**	G4 binding protein (Byrd *et al.*, 2016)	RNP granules (Tian *et al.*, 1991)
12.	**RBM14**	G4 binding protein (von Hacht *et al.*, 2014)	RNP granules (Hennig *et al.*, 2015)
13.	**TAF15**	G4 binding protein (Zheng *et al.*, 2020)	RNP granules (Jobert *et al.*, 2009)
14.	**MATR3 (ALS21)**	associates with G_4C_2 repeats that can form G4 (Ramesh *et al.*, 2020)	RNP granules (Mensch *et al.*, 2018)
15.	***C9orf72***	Intronic G_4C_2 repeat expansions form G4 (Haeusler *et al.*, 2014; Grigg *et al.*, 2014)	RNA foci (Gendron *et al.*, 2013)
16.	**SFPQ**	G4 binding protein (Simko *et al.*, 2020)	RNP granules (Cosker *et al.*, 2016; Fukuda *et al.*, 2020)
17.	**G3BP1**	G4 binding protein (He *et al.*, 2021)	RNP granules (Tourrière *et al.*, 2003)
18.	**NEAT1**	This lncRNA contains abundant G4 motifs (Simko *et al.*, 2020)	Paraspeckles (Hutchinson *et al.*, 2007; Clemson *et al.*, 2009; Sunwoo *et al.*, 2009)
19.	**MALAT1 / NEAT2**	This lncRNA contains abundant G4 motifs (Mou *et al.*, 2021)	Paraspeckles (Hutchinson *et al.*, 2007)

1. SETX: Senataxin, **2.** Zfp106: Zinc finger protein 106, **3.** FUS / TLS: Fused in sarcoma / Translocated in

liposarcoma, **4.** ANG: Angiogenin, **5.** TDP-43: TAR DNA-binding protein of 43 kDa, **6.** ATXN2: Ataxin-2, **7.** hnRNPA1: Heterogeneous nuclear ribonucleoprotein A1, **8.** hnRNPA3: Heterogeneous nuclear ribonucleoprotein A3, **9.** hnRNPA2/B1: Heterogeneous nuclear ribonucleoprotein A2/B1, **10.** EWSR1 / EWR: Ewing sarcoma breakpoint region 1 protein, **11.** TIA1: T-cell intracellular antigen 1, **12.** RBM14: RNA binding motif protein 14, **13.** TAF15: TATA-box binding protein associated factor 15, **14.** MATR3: Matrin3, **15.** C9orf72: chromosome 9 open reading frame 72, **16.** SFPQ: Splicing factor proline and glutamine rich, **17**. G3BP1: GAP SH3 Binding Protein 1, **18.** NEAT1: Nuclear enriched abundant transcript 1, **19.** MALAT1 / NEAT2: Metastasis associated in lung adenocarcinoma transcript-1 / Nuclear enriched abundant transcript 2

Supplementary Table S2.

Synthetic oligonucleotides used for this study (GeneDesign, Inc., Osaka, Japan).
Capital letters indicate deoxyribonucleotide and small letters indicate ribonucleotide.

**Oligos**	**Nucleotide sequence (from 5' to 3')**	
PSD95 G4 dA$_{16}$	ggggaaaagggagggaugggAAAAAAAAAAAAAAAA	(Ishiguro *et al.*, 2016)
CaMKIIα G4 dA$_{16}$	ugggggggcgggugggaugggaAAAAAAAAAAAAAAAA	(Ishiguro *et al.*, 2016)
PSD-95 G4	Ggggaaaagggagggauggg	(Ishiguro *et al.*, 2021)
Cy3-PSD-Ax$_{647}$	Cy3-ggggaaaagggagggauggg-Ax$_{647}$	(Ishiguro *et al.*, 2021)
CaMKIIα G4	ugggggggcgggugggauggga	(Ishiguro *et al.*, 2021)
Cy3-CaMKII-Ax$_{647}$	Cy3-ugggggggcggguggga-Ax$_{647}$	(Ishiguro *et al.*, 2021)
UG$_{10}$-Ax$_{647}$	ugugugugugugugugugug-Ax$_{647}$	(This study)

Supplementary References

Salvi, J. S., & Mekhail, K. R-loops highlight the nucleus in ALS. *Nucleus* **6**, 23–29 (2015).

Marmor-Kollet, H. *et al.* Spatiotemporal Proteomic Analysis of Stress Granule Disassembly Using APEX Reveals Regulation by SUMOylation and Links to ALS Pathogenesis. *Mol. Cell* **80**, 876–891 (2020).

Celona, B. *et al.* Suppression of *C9orf72* RNA repeat-induced neurotoxicity by the ALS-associated RNA-binding protein Zfp106. *eLife* **6**, e19032; 10.7554/eLife.19032 (2017).

Oyoshi, T., & Kurokawa, R. Structure of noncoding RNA is a determinant of function of RNA binding proteins in transcriptional regulation. *Cel. Biosc.* **2**, 1; 10.1186/2045-3701-2-1 (2012).

Imperatore, J. A., McAninch, D. S., Valdez-Sinon, A. N., Bassell, G. J., & Mihailescu, M. R. FUS Recognizes G Quadruplex Structures Within Neuronal mRNAs. *Front. Mol. Biosc.* **7**, 6; 10.3389/fmolb.2020.00006 (2020).

Fujii, R. *et al.* The RNA binding protein TLS is translocated to dendritic spines by mGluR5 activation and regulates spine morphology. *Curr. Biol.* **15**, 587–593 (2005).

Bosco, D. A. *et al.* Mutant FUS proteins that cause amyotrophic lateral sclerosis incorporate into stress granules. *Hum. Mol. Gen.* **19**, 4160–4175 (2010).

Ivanov, P. *et al.* G-quadruplex structures contribute to the neuroprotective effects of angiogenin-induced tRNA fragments. *Proc. Nat. Acad. Sci. USA.* **111**, 18201–18206 (2014).

Emara, M. M. *et al.* Angiogenin-induced tRNA-derived stress-induced RNAs promote stress-induced stress granule assembly. *J. Biol. Chem.* **285**, 10959–10968 (2010).

Molliex, A. *et al.* Phase separation by low complexity domains promotes stress granule assembly and drives pathological fibrillization. *Cell* **163**, 123–133. (2015).

Conicella, A. E., Zerze, G. H., Mittal, J., & Fawzi, N. L. ALS Mutations Disrupt Phase Separation Mediated by α-Helical Structure in the TDP-43 Low-Complexity C-Terminal Domain. *Structure* **24**, 1537–1549 (2016).

Schmidt, H. B., & Rohatgi, R. In Vivo Formation of Vacuolated Multi-phase Compartments Lacking Membranes. *Cell Rep.* **16**, 1228–1236 (2016).

Nonhoff, U. *et al.* Ataxin-2 interacts with the DEAD/H-box RNA helicase DDX6 and interferes with P-bodies and stress granules. *Mol. Biol. Cell* **18**, 1385–1396 (2007).

Sudhakaran, I. P. *et al.* FMRP and Ataxin-2 function together in long-term olfactory habituation and neuronal translational control. *Proc. Nat. Acad. Sci. USA.* **111**, E99–E108; 10.1073/pnas.1309543111 (2014).

Zamiri, B., Reddy, K., Macgregor, R. B., Jr, & Pearson, C. E. TMPyP4 porphyrin distorts RNA G-quadruplex structures of the disease-associated r(GGGGCC)n repeat of the C9orf72 gene and blocks interaction of RNA-binding proteins. *J. Biol. Chem.* **289**, 4653–4659 (2014).

Guil, S., Long, J. C., & Cáceres, J. F. hnRNP A1 relocalization to the stress granules reflects a role in the stress response. *Mol. Cel. Biol.* **26**, 5744–5758 (2006).

Mori, K. *et al.* hnRNP A3 binds to GGGGCC repeats and is a constituent of p62-positive/TDP43-negative inclusions in the hippocampus of patients with C9orf72 mutations. *Act. Neuropath.* **125**, 413–423 (2013).

von Hacht, A. *et al.* Identification and characterization of RNA guanine-quadruplex binding proteins. *Nuc. Aci. Res.* 42, 6630–6644 (2014).

Mori, K. *et al.* Reduced hnRNPA3 increases C9orf72 repeat RNA levels and dipeptide-repeat protein deposition. *EMBO Rep.* **17**, 1314–1325 (2016).

Scalabrin, M. *et al.* The cellular protein hnRNP A2/B1 enhances HIV-1 transcription by unfolding LTR promoter G-quadruplexes. *Sci. Rep.* **7**, 45244; 10.1038/srep45244 (2017).

Martinez, F. J. *et al.* Protein-RNA Networks Regulated by Normal and ALS-Associated Mutant HNRNPA2B1 in the Nervous System. *Neuron* **92**, 780–795 (2016).

Takahama, K., Kino, K., Arai, S., Kurokawa, R. & Oyoshi, T. Identification of Ewing's sarcoma protein as a G-quadruplex DNA- and RNA-binding protein. *FEBS J.* **278**, 988–998 (2011).

Andersson, M. K. *et al.* The multifunctional FUS, EWS and TAF15 proto-oncoproteins show cell type-specific expression patterns and involvement in cell spreading and stress response. *BMC Cell. Biol.* **9**, 37; 10.1186/1471-2121-9-37 (2008).

Byrd, A. K. *et al.* Evidence That G-quadruplex DNA Accumulates in the Cytoplasm and Participates in Stress Granule Assembly in Response to Oxidative Stress. *J. Biol. Chem.* **291**, 18041–18057 (2016).

Tian, Q., Streuli, M., Saito, H., Schlossman, S. F., & Anderson, P. A polyadenylate binding protein localized to the granules of cytolytic lymphocytes induces DNA fragmentation in target cells. *Cell* **67**, 629–639 (1991).

Hennig, S. *et al.* Prion-like domains in RNA binding proteins are essential for building subnuclear paraspeckles. *J. Biol. Chem.* **210**, 529–539 (2015).

Zheng, K. W. *et al.* Detection of genomic G-quadruplexes in living cells using a small artificial protein. *Nuc. Aci. Res.* **48**, 11706–11720 (2020).

Jobert, L., Argentini, M., & Tora, L. PRMT1 mediated methylation of TAF15 is required for its positive gene regulatory function. *Exp. Cel. Res.* **315**, 1273–1286 (2009).

Ramesh, N. *et al.* RNA dependent suppression of C9orf72 ALS/FTD associated neurodegeneration by Matrin-3. *Act. Neuropath. Com.* **8**, 177; 10.1186/s40478-020-01060-y (2020).

Mensch, A. *et al.* The p.S85C-mutation in MATR3 impairs stress granule formation in Matrin-3 myopathy. *Exp. Neuro.* **306**, 222–231 (2018).

Grigg, J. C., Shumayrikh, N., & Sen, D. G-quadruplex structures formed by expanded hexanucleotide repeat RNA and DNA from the neurodegenerative disease-linked C9orf72 gene efficiently sequester and activate heme. *PloS one* **9**, e106449; 10.1371/journal.pone.0106449 (2014).

Gendron, T. F. *et al.* Antisense transcripts of the expanded C9ORF72 hexanucleotide repeat form nuclear RNA foci and undergo repeat-associated non-ATG translation in c9FTD/ALS. *Act. Neuropath.*

126, 829–844 (2013).

Simko, E. *et al.* G-quadruplexes offer a conserved structural motif for NONO recruitment to NEAT1 architectural lncRNA. *Nuc. Aci. Res.* **48**, 7421–7438 (2020).

Cosker, K. E., Fenstermacher, S. J., Pazyra-Murphy, M. F., Elliott, H. L., & Segal, R. A. The RNA-binding protein SFPQ orchestrates an RNA regulon to promote axon viability. *Nat. Neurosci.* **19**, 690–696 (2016).

Fukuda, Y. *et al.* Binding and transport of SFPQ-RNA granules by KIF5A/KLC1 motors promotes axon survival. *J. Biol. Chem.* **220**, e202005051; 10.1083/jcb.202005051 (2021).

He, X., Yuan, J., & Wang, Y. G3BP1 binds to guanine quadruplexes in mRNAs to modulate their stabilities. *Nuc. Aci. Res.* **49**, 11323–11336 (2021).

Tourrière, H. et al. J. The RasGAP-associated endoribonuclease G3BP assembles stress granules. *J Cell Biol*. **160**, 823–831 (2003).

Hutchinson, J. N. *et al.* A screen for nuclear transcripts identifies two linked noncoding RNAs associated with SC35 splicing domains. *BMC Genom.* **8**, 39; 10.1186/1471-2164-8-39 (2007).

Clemson, C. M. *et al.* An architectural role for a nuclear noncoding RNA: NEAT1 RNA is essential for the structure of paraspeckles. *Mol. Cell* **33**, 717–726 (2009).

Sunwoo, H. *et al.* MEN epsilon/beta nuclear-retained non-coding RNAs are up-regulated upon muscle differentiation and are essential components of paraspeckles. *Gen. Res.* **19**, 347–359 (2009).

Mou, X., Liew, S. W., & Kwok, C. K. Identification and targeting of G-quadruplex structures in MALAT1 long non-coding RNA. *Nuc. Aci. Res.* **50**, 397–410 (2022).
